# Comparative Transcriptome Analysis of Gene Expression Patterns in Tomato Under Dynamic Light Conditions

**DOI:** 10.3390/genes10090662

**Published:** 2019-08-29

**Authors:** Juanjuan Ding, Jiantao Zhao, Tonghua Pan, Linjie Xi, Jing Zhang, Zhirong Zou

**Affiliations:** 1 College of Horticulture, Northwest A&F University, Yangling 712100, China; 2 INRA, UR1052, Génétique et Amélioration des Fruits et Légumes, Domaine Saint Maurice, 67 Allée des Chênes CS 60094, 84143 Montfavet, France

**Keywords:** RNA sequencing, *Solanum lycopersicum*, different light regimes, differentially expressed genes, photosynthesis

## Abstract

Plants grown under highly variable natural light regimes differ strongly from plants grown under constant light (CL) regimes. Plant phenotype and adaptation responses are important for plant biomass and fitness. However, the underlying regulatory mechanisms are still poorly understood, particularly from a transcriptional perspective. To investigate the influence of different light regimes on tomato plants, three dynamic light (DL) regimes were designed, using a CL regime as control. Morphological, photosynthetic, and transcriptional differences after five weeks of treatment were compared. Leaf area, plant height, shoot /root weight, total chlorophyll content, photosynthetic rate, and stomatal conductance all significantly decreased in response to DL regimes. The biggest expression difference was found between the treatment with the highest light intensity at the middle of the day with a total of 1080 significantly up-/down-regulated genes. A total of 177 common differentially expressed genes were identified between DL and CL conditions. Finally, significant differences were observed in the levels of gene expression between DL and CL treatments in multiple pathways, predominantly of plant–pathogen interactions, plant hormone signal transductions, metabolites, and photosynthesis. These results expand the understanding of plant development and photosynthetic regulations under DL conditions by multiple pathways.

## 1. Introduction

Global agriculture faces an increasing demand due to growing population, climate change, and constraints of land, water, and rural farm labors [1]. The light source is the main factor that directly impacts crop yield and crop performance by influencing photosynthesis and light-signaling metabolism processes. Agricultural production and the achieved crop quality can be influenced by many light-related factors, such as light intensity [2,3], light quality [4,5,6], light period [7,8], and light source [9,10]. In the field, plants experience fluctuating sunlight conditions due to diurnal variations of light intensity, temporary shading by clouds, neighboring plants [11], as well as the movement of leaves and branches by wind [12]. Artificial light sources are widely used in modern crop cultivation systems, especially in a controlled environment, to increase the yield of agriculture products. Adjusting artificial light resource plays an important role in improving high-efficient productions of major crops. Lettuce fresh weight was shown to increase with light intensity level, except for the 800 µmol m^-1^ s^-1^ treatment [2,3]. Many previous studies agree that red and blue light play an important role in yield [4,5,6]. 

Many researches have focused on the impact of the difference between constant light (CL) and fluctuating light (FL) conditions on plants [13,14,15,16]. For existence, the duration of light intensity switch can by ranged from seconds and minutes to hours [12,17]. The duration of light intensity period of common rays of sunlight upon the canopy of plants in natural conditions usually changes rapidly and irregularly [16,18,19,20,21,22]. 

When plants receive the same daily light integral (DLI), photosynthesis can be significantly different under different light regimes due to different aspects: (1) The conversion efficiency of leaf photosynthesis decreases with increasing light intensity at specific light spectra [23]. (2) The light energy absorbed by the chloroplast increases, and the excess light energy could damage the plants. Plants develop different photo protective mechanisms [24], such as non-photochemical quenching (NPQ) in the chloroplast thylakoid membrane, light-harvesting complexes (LHCs), and de-epoxidantion of zeaxanthin [25,26,27]. (3) The speed of opening stomata is lower than the initial up-regulation of photosynthetic electron transport, leading to an insufficient supply of CO_2_ for the carbon cycle during transitions [16]. (4) The rate of enzyme activation in the Calvin cycle also limits photosynthesis under FL conditions [16,28]. Plants grown under FL conditions have thinner leaves [20,21] and smaller total leaf areas [12,20]. Interestingly, the responses of genes related to photosynthesis and vitamin metabolism are different between dynamic light duration occurring in the morning or at the end of the day. This shows that the circadian clock and the dynamic light signal work together and modulate related gene expressions during acclimation responses [29].

Compared with photoprotection and biochemical properties of leaves acclimated under dynamic conditions, much less is known about gene expression of acclamatory process. Our knowledge in the morphological, physiological, and transcriptional regulations of tomato plants under DL conditions is still limited. The question remains whether gene expressions and physiology characteristics remain the same when the period of light intensity changes during a day.

In this study, we designed three different light intensity distributions and investigated their effects on the tomato plant’s morphological, physiological, and transcriptional levels during the early development stages of the tomato plants. This study provides useful knowledge for the improvement of both light-use efficiency and yield by using light source adjustments.

## 2. Materials and Methods 

### 2.1. Materials and Plant Growth Condition

Tomato seeds (*Solanum lycopersicum*, Jinpeng No.1) were used as research material. Seeds were sown in a plastic seedling tray (53 × 27.5 × 4.5 cm) filled with substrate (Pindstrup, Demark) within the artificial climate chamber at south campus, Northwest A&F University, Yangling, China. The pH of the nutrient solution was 5.5, with the concentration of N, P2O5, and K2O at 28, 76, and 132 mg/L, respectively. The temperature and relative humidity during the time were 28 °C and 65%, respectively, which decreased to 18 °C and 55% at night. Each group contained 144 seeds at the beginning of the experiment. Three weeks after sowing, 60 uniform seedlings with two fully expanded leaves were transplanted into 7 × 7 × 8 cm black plastic pots filled with substrate (Pindstrup, Demark). Then, plants were set 10–13 cm from each other. After three weeks’ irrigation with a half dose Yamazaki nutrient solution (EC 1.0 ± 0.2 mS/cm), the dose of the solution was doubled (pH 6.5 ± 0.5, EC 2.0 ± 0.5 mS/cm) until the end of the experiment. The treatments were conducted after seedlings unearth using fluorescent light (CFLS; TL 5 Essential 21W/ 865, Philips, Shanghai, China), and the lighting array was fixed at 10 cm above the plant canopy. Plants were moved every three days at random to take into account any heterogeneity in the light intensity. 

### 2.2. Light Intensity Distribution Design

**Figure 1:**
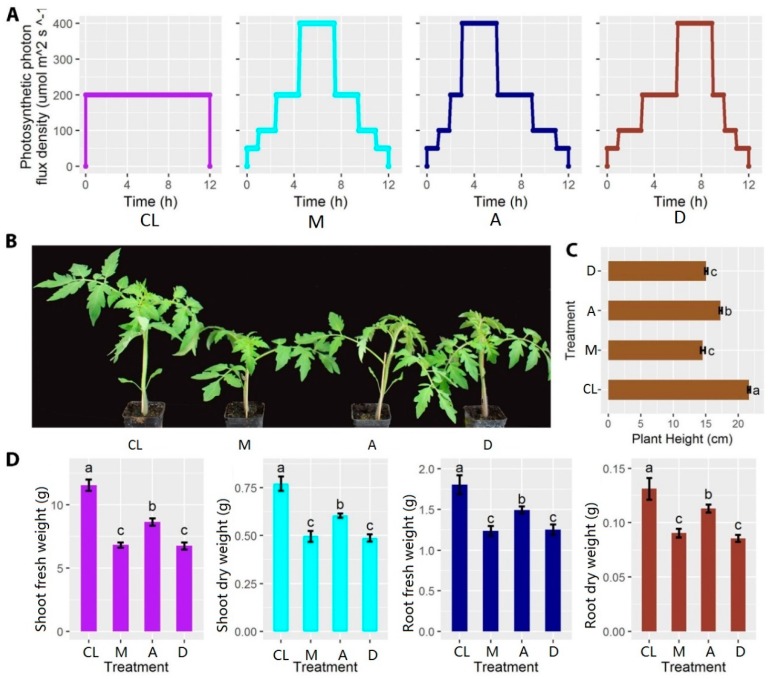
Experimental design and measurements of fresh and dry weight of shoots and roots, respectively. (**A**) Light distribution patterns of control (CL), treatment 1 (M), treatment 2 (A), and treatment 3 (D). (**B**) Morphological comparisons between different treatments after five weeks (*n* = 8). (**C**) Comparison of plant height between different treatments after five weeks. (**D**) Comparisons of shoot fresh weight, shoot dry weight, root fresh weight, and root dry weight between different treatments after five weeks. Different lowercase letters indicate a significant difference among the same treatments (*p* < 0.05). Each bar represents the mean ± SE (standard error) of eight biological replicates.

Three sinusoid types of dynamic light regimes with different phase positions over a day (treatments M, A and D) were designed to investigate their effects on the early-stage development of tomato plants. DL regimes were compared to a constant light condition, while the DL models were less dynamic compared to those experienced by crops grown in the field. The total daily light integral for each treatment and the control was the same during each day, with a total lighting period of 12 h. First, a constant light intensity (CL) of 200 μmol m^-1^ s^-1^ was used for 12 h as control. The highest light intensity was 400 μmol m^-1^ s^-1^ with a phase position at midday (M), advanced by 1.5 h compared with M (A), and delayed by 1.5 h compared with M (D) (Figure 1A). Light intensity was measured using the PAR meter (Model MQ-100, Apogee Instruments Inc., Logan, UT, USA). The nutrient solution and environmental conditions, except the light regimes, were the same until the end of the experiment.

### 2.3. Morphological Measurements

After five weeks of light treatments, fresh tomato plants were randomly divided into two groups. The fresh developed leaves from the first group with similar morphological shapes were immediately frozen with −80 °C liquid nitrogen for RNA sequencing. The remaining samples were used for measurements of some morphological traits, including plant height, shoot/ root fresh weight, which were quickly dried at 105 °C for 15 min and then kept at 60–80 °C for 48 h, until the samples were completely dried. The shoot/ root dry weight were then measured to compare with the fresh weight. Leaf areas were measured using the scanner (EPSON PERFECTION V700 PHOTO, Epson (China) Co., Ltd., Beijing, China). Eight plants of each treatment were selected for growth characteristic analysis. 

### 2.4. Analysis of Gas Exchange Parameters and Chlorophyll Concentration

All gas exchange and chlorophyll concentrations were measured on the fourth to fifth fully expanded leaf (counted from bottom of the plant), after five weeks of light treatments. All photosynthetic gas exchange was measured using a Li-cor 6400XT portable gas exchange system (LI-6400, LI-COR Inc., Lincoln, NE, USA) with a transparent leaf chamber. Measurements were conducted between 08:00 am and 20:00 pm. For the measurements, 10 plants were selected and two Li-cors were used, with measurements of photosynthetic parameters including net photosynthetic rate (Pn) and stomatal conductance (Gs) recorded every hour. The relative humidity in the assimilation chamber was maintained at 60–80%; the leaf temperature in the measurement chamber was maintained at 20 °C; the external CO_2_ concentration was 400 ± 20 μmol mol^-1^; the light intensity was measured according to the light treatment.

Seven plants were selected for chlorophyll concentration analysis. The weighed fresh leaf tissue (0.1 g) was extracted in 96% alcohol/water (v/v). The extract was centrifuged (H2050R; Xiang Yi Centrifuge instrument, Co., Ltd., Changsha, China) at 10,000 *g* for 10 min. The supernatant was separated, and the absorbance was measured at 400–700 nm using a spectrophotometer (UV-1800; Shimadzu Co., Kyoto, Japan) at wavelengths of 665 nm (A665), 649 nm (A649), and 470 nm (A470), respectively. The chlorophyll a, chlorophyll b, and total chlorophyll concentrations were measured by spectrophotometry and calculated according to the following equations of Lichtentaler and Wellburn [30]: Chlorophyll a concentration = (13.95 × A665 – 6.88 × A649) × 20/(1000 × 0.1), chlorophyll b concentration = (24.96 × A649 –7.32 × A665) × 20/(1000 × 0.1), and total chlorophyll concentrations = chlorophyll a concentration + chlorophyll b concentration. Chl a/b = chlorophyll a concentration/ chlorophyll b concentration.

### 2.5. RNA Extraction and Illumina Sequencing

Total RNAs were extracted from the frozen fresh tomato leaves using the EASYspin Plus Kit according to the manufacturer’s instructions (Aidlab Biotechnologies Co. Ltd., Beijing, China). The quality and quantity of extracted RNAs was measured using agar gel electrophoresis and Nanodrop micro spectrophotometer (Thermo Scientific, Wilmington, DE, USA). RNAs from three biological repeats (0.5 g per sample) from at least five plants with the same concentration and volume were equally combined for RNA-seq. Library was constructed using the NEBNext Ultra RNA library prep kit (NEB#E7530, New England Biolabs, Ipswich, MA, USA). The quality of the cDNA library was measured using DNA 1000 assay Kit (5067-1504, Agilent Technologies, Santa Clara, CA, USA) before sequencing on an Illumina HiSeq TM 2500 by Gene Denovo Biotechnology Co. (Guangzhou, China).

### 2.6. Sequence Quality Control and De Novo Assembly

Raw reads containing adapters with more than 10% of unknown nucleotides and with more than 50% of low quality (Q-value ≤ 20) bases were filtered before mapping to ribosome RNA (rRNA) database in Bowtie2 [31]. Mapped rRNA reads were removed before mapping to reference genome by TopHat2 (version 2.0.3.12) [32]. The reconstruction of transcripts was carried out with software Cufflinks [33], together with TopHat2. Gene abundances were quantified by software RSEM [34]. The gene expression level was normalized by using the FPKM (Fragments Per Kilobase of transcript per Million mapped reads) method. Single-nucleotide polymorphism (SNP) was identified in GATK [35] and SNP/InDel annotation was done using ANNOVAR [36].

### 2.7. Differentially Expressed Genes (DEGs) Analysis

Differentially expressed genes across treatments and control were identified using the edgeR package (http://www.r-project.org/) in R. Genes with a fold change ≥2 and a false discovery rate (FDR) <0.05 were treated as significant DEGs. DEGs were then subjected to enrichment analysis of GO functions and KEGG pathways.

Gene Ontology (GO) enrichment analysis provides all GO terms that are significantly enriched in DEGs compared to the genome background. All DEGs were mapped to GO terms in the Gene Ontology database (http://www.geneontology.org/). Significantly enriched GO terms (FDR correction *p*-value ≤ 0.05) were identified by hypergeometric test by comparing with the genome background. Pathway enrichment analysis was performed using the Kyoto Encyclopedia of Genes and Genomes (KEGG) database [37]. Pathways with FDR-corrected *p*-values ≤ 0.05 were defined as significantly enriched pathways in DEGs.

## 3. Results

### 3.1. Experimental Design and Phenotypic Characterization

After five weeks of treatment, plant heights of all three treatments were significantly lower compared with the control (Figure 1B, C). In addition, both the fresh and dry weight of shoots and roots were also significantly reduced, compared with the control (Figure 1D). Interestingly, among DL treatments, A showed a smaller difference compared with control with regard to plant height and biomass, but M and D treatments showed a bigger difference. These results indicate that the light intensity distributions during the day exerted a significant impact on the morphological development of tomato plants.

### 3.2. Changes in the Photosynthetic Characteristics of the Leaves 

Photosynthesis is an extremely important metabolic process in plants. The average value of the daily photosynthetic rate (Pn) of DL treatments was slightly lower than that of the CL condition. Plants grown under treatment D had the lowest value of Gs. Chlorophyll content is an important indicator of plant growing conditions and photosynthetic capacity. Plants grown under DL treatments displayed significantly lower total chlorophyll content compared with plants grown under the CL condition. Plants grown under DL conditions had significantly higher chlorophyll a/b ratios compared with plants grown under the CL condition. In addition, plants grown under M and D conditions had the smallest leaf areas compared with plants grown under other treatments. The leaf area of the M treatment was 17% less than that of the CL treatment (Table 1). The results of the photosynthetic characteristics indicate that the plants grown under DL conditions had lower photosynthetic capability and light capture area compared with plants grown under the CL condition.

### 3.3. Transcriptome Sequencing, Assembly, and Annotation

**Figure 2:**
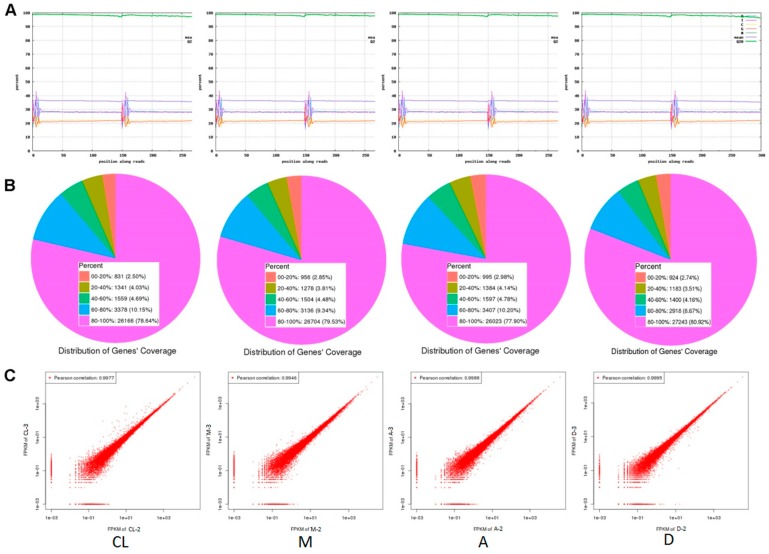
Transcriptome sequencing quality analysis between treatments and control. (**A**) Base composition and quality distributions. (**B**) Statistic maps of gene coverage. (**C**) Correlation coefficient maps between different sequencing repeats.

To understand the mechanisms of the effects of light intensity distributions on the development of tomato plants, RNA-seq was performed based on deep transcriptome sequencing analysis after five weeks of treatments. The sequencing quality for all the treatments and control was quite high, after discarding the raw sequencing data. The percentage of bases with Q20 (high sequencing quality) was close to 100% (Figure 2A). Gene coverage ranged from 80 to 100%, accounting for approximately 80% of the total genes (Figure 2B). Within each control or treatment, the correlation coefficient between replicates exceeded 99.5%, indicating high consistency between replicates (Figure 2C). Summaries of raw sequence quality before and after filtering and the number of reads mapped to the reference genome (version 3.0) are provided in Supplementary Table S1. These results show that the transcriptome sequencing quality was sufficient for further analyses. 

### 3.4. Gene Expression Difference Analysis

**Figure 3:**
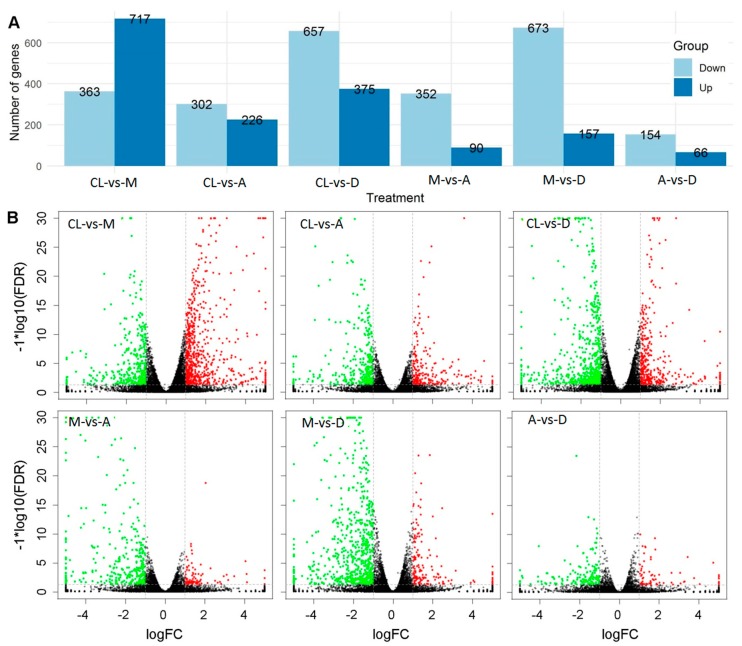
Difference analysis of gene expression between treatments. (**A**) Comparison of the number of up- and down-regulated genes. (**B**) Volcano plots between treatments and control. Red and green points represent up- and down-regulated genes, respectively. Block points represent no difference genes.

Differentially expressed genes (DEGs) between treatments and control were identified using edgeR software [38,39]. FDR and log2FC were used to screen for DEGs. The screening conditions were FDR < 0.05 and |log2FC| > 1. Hundreds of genes were up- or down-regulated between control and treatments, as well as between different treatments (Supplementary Tables S2–S8). The total number of significantly regulated genes differed in different comparisons (Figure 3A). The largest difference was found between treatment M and control (1080 significant up-/down-regulated genes), followed by treatment D and control (1032 significant up-/down-regulated genes). In general, the number of up-regulated genes was lower than that of down-regulated genes, with the only exception between treatment M and control. In particular, the largest difference between the numbers of down-regulated and up-regulated genes was found between treatments M and D, reaching a total of 416 genes (Figure 3A). The smallest number of significantly regulated genes was found between treatment A and D, with 66 and 154 significantly up- and down-regulated genes, respectively. Volcano plots show that the number of up-and down-regulated genes had a distinct distribution pattern between the three DL treatments and control (Figure 3B). For example, the distribution pattern of down-regulated genes of treatment A was much higher than the patterns of treatments A and D, respectively. Although the number between down- and up-regulated genes was low between treatments A and D compared with other treatments, the distribution patterns were quite similar (Figure 3B). These results show clear global gene expression patterns between different treatments and control.

### 3.5. DEGs GO/Pathway Enrichment Analysis

**Figure 4:**
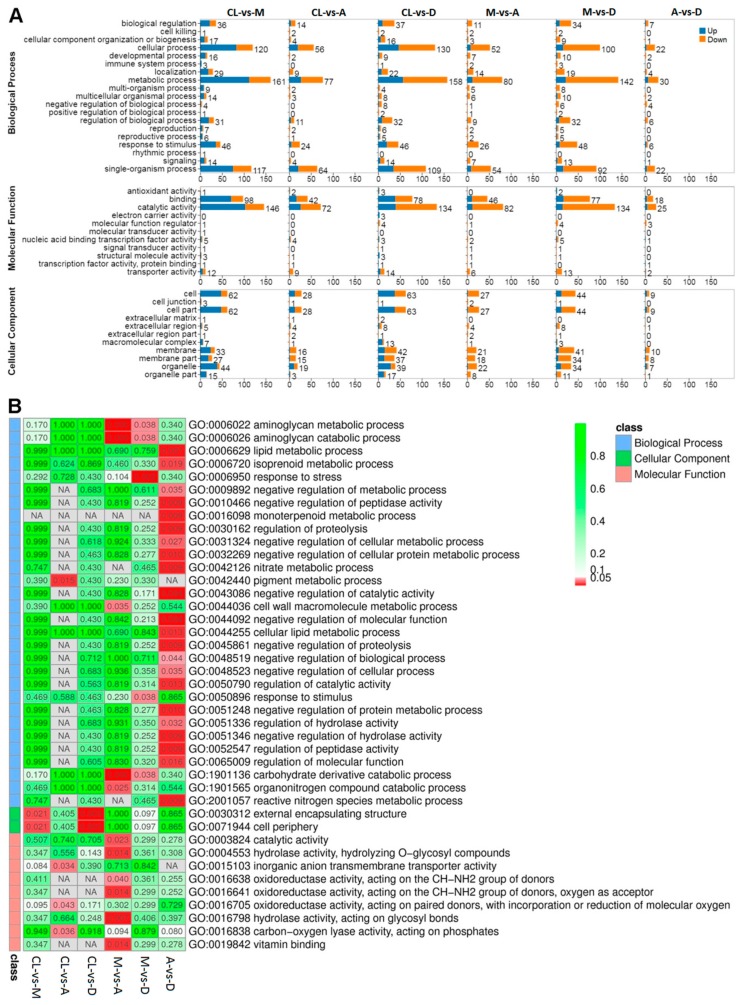
DEGs GO/Pathway enrichment analyses between different light regimes. (**A**) Summary of the distribution and number of DEGs in three ontology classes, including molecular function, cellular component, and biological process. (**B**) Q-value heatmap of the GO pathway enrichment of the three main ontology classes. The color scale indicates the Q-value. Darker coloration indicates more significant enrichment.

GO and pathway enrichment analyses were performed for all significant DEGs (Figure 4). Different comparisons show similar distribution patterns with regard to the numbers and types of enriched pathways, which can be divided into three main functional groups, including 19 biological processes, 11 molecular functions, and 11 cellular components (Figure 4A). However, the enrich level (Q-value) for each functional group varied (Figure 4B). The Q-value is the *p*-value after multiple hypothesis test corrections, which ranges from 0 to 1. The closer it is to zero, the more significant the enrichment. Notably, most of the functional groups that were significantly enriched were involved in different cellular metabolic pathways, such as monoterpenoid, cellular, nitrate, and pigment metabolic processes (Figure 4B).

### 3.6. KEGG Enrichment Analysis

**Figure 5:**
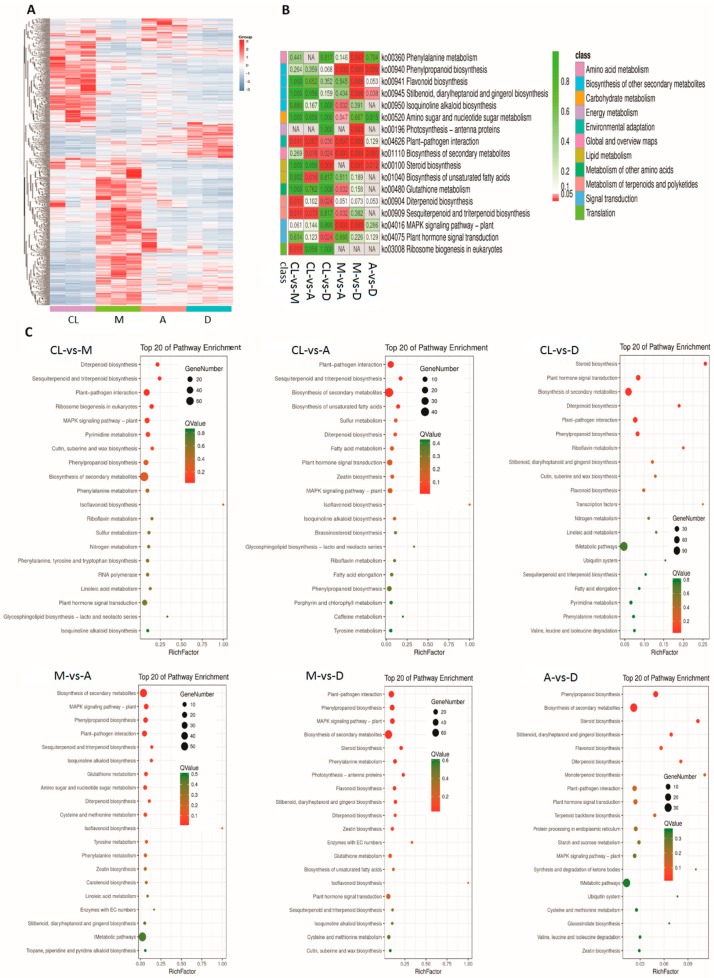
Gene expression patterns between DL treatments and control and KEGG enrichment analysis. (**A**) Global gene expression patterns between control and treatments. (**B**) Q-value heatmap of KEGG enrichment. (**C**) KEGG pathway enrichment analysis between control and DL treatments. RichFactor refers to the ratio of the number of transcripts in the pathway entry in the differentially expressed transcript to the total number of transcripts in the transcript that are located in the pathway entry. The larger the RichFactor, the higher the degree of enrichment is. The dot size indicates the number of DEGs of the pathway, and the dot colour indicates the Q-value.

The gene clustering heatmap shows a distinct global gene expression pattern between control and DL treatments (Figure 5A). The expression patterns of most DEGs under treatments were completely opposite. Most of the genes with higher expression levels under CL had lower expression levels under DL, and vice versa. Moreover, most of the DEGs showed large differences in expression profiles under the three DL treatments. KEGG expression enrichment analysis (Figure 5C) shows that in the comparison of DL treatments versus CK, DEGs were most highly enriched in plant–pathogen interaction, plant hormone signal transduction, diterpenoid biosynthesis, sesquiterpenoid and triterpenoid biosynthesis, phenylpropanoid biosynthesis, and biosynthesis of secondary metabolites. In DL comparisons, DEGs were most highly enriched in plant–pathogen interaction, the MAPK signaling pathway, phenylpropanoid biosynthesis, diterpenoid biosynthesis, and biosynthesis of secondary metabolites. In addition, the Q-value of the KEGG enrichment indicates that the largest number of enriched genes was involved in the biosynthesis of secondary metabolites, although the degree of enrichment might not be the highest compared with the other top enriched pathways (Figure 5B).

### 3.7. Identification of Common DEGs Under Dynamic Light Conditions 

**Figure 6:**
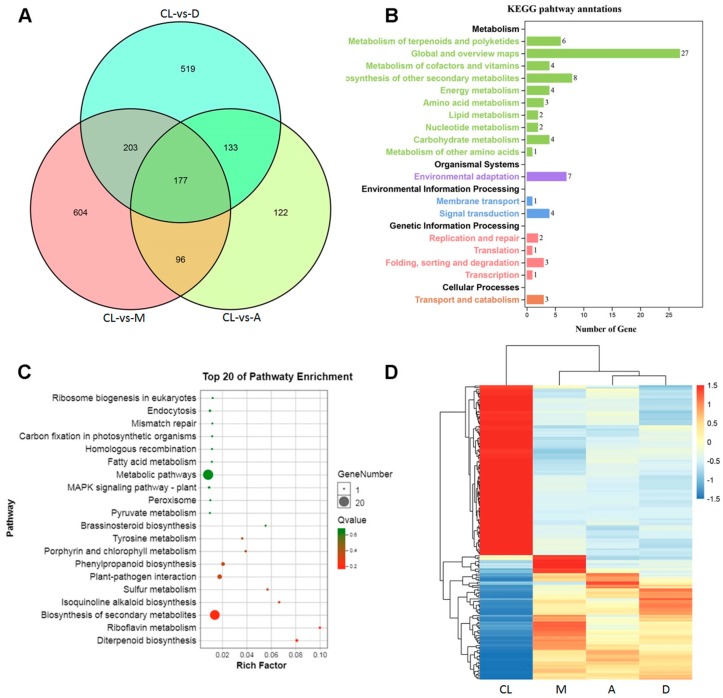
Common DEGs under fluctuating light treatments. (**A**) Venn diagram of common genes detected in three DL treatments compared with the CL treatment. (**B**) KEGG pathway enrichment for the 177 common genes. (**C**) Top 20 enriched KEGG pathways. (**D**) Hierarchical clustering of common genes based on normalized FPKM values under the four tested light regimes. Blue indicates lower expression, and red indicates higher expression.

In this study, three out of four treatments were characterized with DL conditions. We further quantified the common DEGs compared with the CL (Figure 6), and 177 common DEGs were identified (Figure 6A). KEGG pathway annotation showed that these genes were involved in different metabolic processes, such as terpenoids, vitamins, amino acids, and lipids (Figure 6B). Among the top 20 most enriched pathways, biosynthesis of metabolic pathways represented the most significantly enriched (Figure 6C). In addition, a distinct correlation pattern was found between the CL condition and DL conditions (Figure 6D). Although differences among different DL treatments were also identified, the differences were less important compared with that of the CL condition (Figure 6D). 

### 3.8. Specific Up- and Down-Regulated Genes

**Figure 7:**
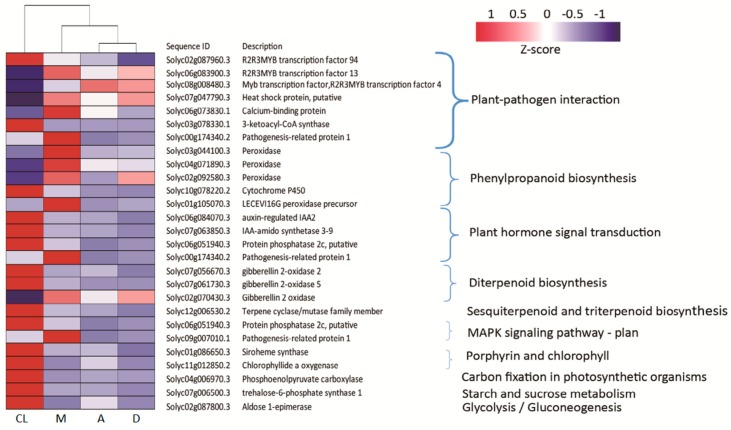
Heatmap of transcriptional levels for specific up- and down-regulated 27 DEGs enriched in DL compared with CL. In this heatmap, the columns represent tomato leaf samples treated with different light regimes, and the rows represent DEGS enriched in 10 KEGG pathways. Relative expression levels were normalized based on the Z-score and are shown as a color gradient from low (blue) to high (red).

The tomato plant development is a very complex process. The RNA-seq data shows that common DEGs were involved in many relevant pathways where they modulate metabolic processes, such as the plant–pathogen interaction, phenylpropanoid biosynthesis, plant hormone signal transduction, biosynthesis of terpenoids, and photosynthetic metabolic processes (Figure 7). Three of the DEGs involved in plant –pathogen interaction belonged to the R2R3-MYB transcription factor family, which is involved in stress responses and biosynthesis of secondary metabolites. In particular, one gene (Solyc02g087960.3) was down-regulated and two genes (Solyc06g083900.3 and Solyc08g008480.3) were up-regulated compared with the CL. In addition, *heat shock protein* (Solyc07g047790.3), *calcium-binding protein* (Solyc06g073830.1), and *pathogenesis-related protein 1* (Solyc00g174340.2) were all up-regulated in response to M treatment. However, *3-ketoacyl-coA synthase* (Solyc03g078330.1) was down-regulated under all DL treatments. 

With regard to the DEGs involved in the phenylpropanoid biosynthesis, four peroxidase-related genes were detected which were involved in the biosynthesis of lignin. Solyc03g044100.3, Solyc04g071890.3, Solyc02g092580.3, and *LECEVI16G peroxidase precursor* were all up-regulated in respond to M treatment. In contrast, *cytochrome P450* (Solyc10g078220.2), which played an important role in preventing plant injury, was down-regulated under DL treatment. For DEGs involved in plant hormone signal transduction, four genes, including two IAA-regulated genes (Solyc06g084070.3 and Solyc07g063850.3) and one ABA-regulated gene (Solyc06g051940.3), showed decreased expression in response to DL treatment. In addition, one terpene synthesis-related gene showed decreased expression in DL treatments, except for the Gibberellin 2 oxidase gene (Solyc12g0006530.2). 

### 3.9. SNP/InDel Annotations

**Figure 8:**
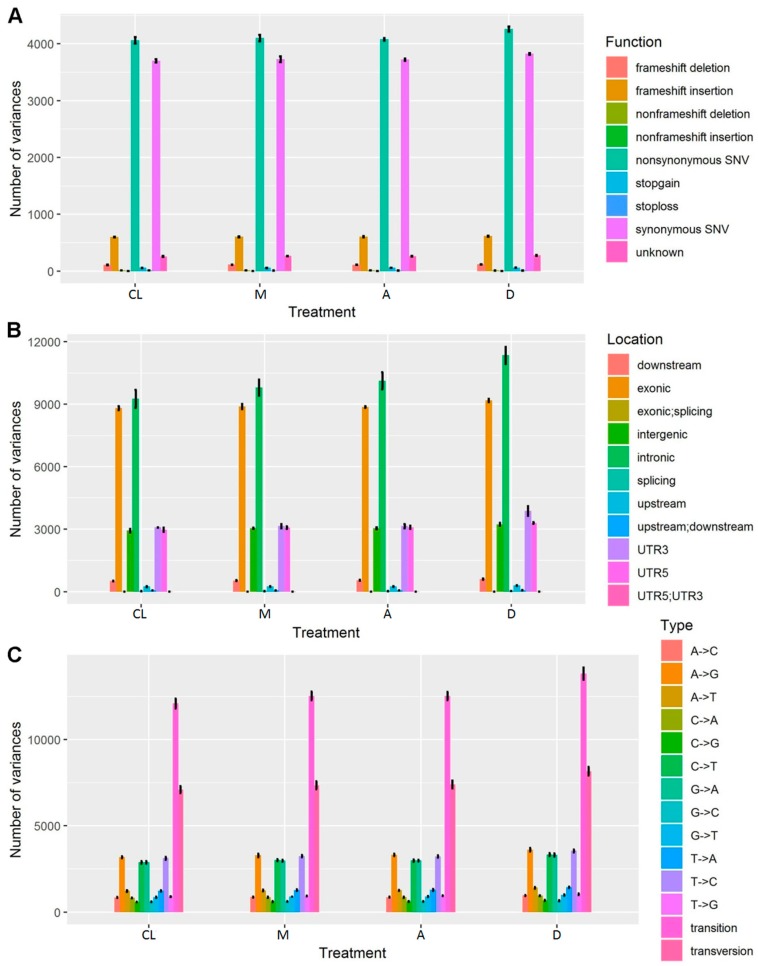
SNP/InDel Annotations in terms of function (**A**), location (**B**), and type (**C**).

Transcriptome sequencing also identified various single nucleotide polymorphisms (SNPs) (Figure 8). Up to nine types of functional variations were identified for each control and DL treatment, such as frameshift/nonframeshift deletion/insertion, and synonymous/nonsynonymous single nucleotide variants (SNV) (Figure 8A). Among these, nonsynonymous SNV and synonymous SNV represented dominant functional variations, with overall similar trends for all types of functional variations (Figure 8A). In addition, these SNPs were located in different locations, with dominant locations in exonic and intronic locations, both of which were highest between all DL treatments and control (Figure 8B). All mutations were identified in each treatment, with transition and transversion as the two dominant types (Figure 8C). These results demonstrate a comprehensive transcriptional regulation in tomato under different light intensity regimes.

## 4. Discussion

### 4.1. Dynamic Light Affects the Growth and Photosynthetic Characteristics of Tomato Plants

The light environment acts not only as a photosynthetic driving force, but also as a signal for plant morphological and physiological adaptions in response to different environmental changes [40,41,42,43,44,45]. Plants experience constantly changing light conditions under the natural environment. A more recent study explored the responses of photosynthesis [13,15,16,28,46] and morphology [21] in response to DL conditions. A better understanding of the physiological, photosynthetic, and transcriptional responses to DL may provide a new stimulus to improve photosynthesis for crop growth in the field. 

Under DL regimes, photosynthesis rates were lower compared with plants grown under CL. The major reason was that the efficiency of radiation declined with increasing light intensity. The efficiency of radiation use was high under low light. Under CL treatment, the light intensity always remained at 200 μmol m^-2^ s^-1^, while the efficiency was low under 400 μmol m^-2^ s^-1^ light period, and the quantum yield of CO_2_ fixation was reduced under DL treatments. Furthermore, changes in photosynthesis-related enzyme activities also regulated photosynthesis [47,48]. This study showed that the daily photosynthetic rate was lower under DL conditions compared to CL regime, and stomatal conductance followed the same trend (Table 1). However, in this experiment, the photosynthesis parameters were measured only once per hour, thus losing much transient changes between two measurement points. In addition, the chlorophyll content also decreased under DL treatments, while chlorophyll a/b increased, which is consistent with previous studies [12,17,20]. 

When plants are grown under high light conditions, they accumulate less chlorophyll content and have smaller light-harvesting antennae compared with plants in low light conditions [49,50,51]. The down-regulated chlorophyll content prevents the excess light from damaging the photosynthetic metabolic process, which enhanced plant fitness under DL conditions. The results of this study show that plants grown under DL condition had a less-expanding leaf area, which is consistent with previous research [12,20,21]. Results from this study also demonstrated that DL inhibited plant height. Yang (2018) showed that light intensity played a vital role in regulating soybean seedling height and leaf morphology. Previous studies also reported that leaf morphology [52] and stem elongation [53] were significantly affected by reduced light intensities. This finding suggests that DL had the same effect in response to high light to plant morphology and physiology.

The plant performance under DL had disadvantages to maximize the light utilized for carbon fixation. In brief, plants grown under CL regime had higher photosynthetic capacity and larger leaf area to fully utilize the absorbed light for carbon fixation, which resulted in a higher dry mass compared with plants grown under DL conditions. In this experiment, the distribution of different light periods affected the growth and development of the tomato plants. Biomass was significantly lower under M and D treatments compared with A treatment (Figure 1). This interesting phenomenon can be explained by the time of day, which possibly regulates the expression of related genes, such as genes related to the circadian clock, to affect photosynthesis and plant hormone signal, thus ultimately affecting plant morphology and biomass. In general, many genes that are involved in the hormone metabolism are regulated in a circadian pattern. Abscisic acid, auxin, and cytokinins are strongly regulated by the circadian clock [54]. Additionally, photosynthesis-related genes are also regulated by the circadian clock [55].

### 4.2. Transcriptional Regulations in Response to Dynamic Light

A highly variable light environment changes the plant performance and regulates related gene expressions to improve the fitness in the field [29]. The obtained data showed transcriptome features and identified candidate genes that are likely responsible for plant adaption to different light regimes at different levels. The results not only provide useful information to predict gene expression in tomato plants grown in the field, but also help to understand the transcriptional regulation of plant developmental plasticity. 

In this study, based on KEGG and GO pathway annotations, under DL conditions, DEGs were most highly enriched in plant–pathogen interaction, plant hormone signal transduction, diterpenoid biosynthesis, sesquiterpenoid and triterpenoid biosynthesis, phenylpropanoid biosynthesis, and biosynthesis of secondary metabolites (Figure 7 and Figure 8). 

Plant–pathogen interactions. The R2R3MYB proteins form one of the largest families of transcription factors and play a crucial role in developmental processes [56,57] and responses to biotic and abiotic stresses [58,59,60]. In this study, three genes related to R2R3MYB transcription factors were identified, the expression levels of R2R3MYB transcription factor 13 (Solyc06g083900.3) and R2R3MYB transcription factor 4 (Solyc08g008480.3) were more highly expressed under DL conditions than the CL condition. Under cold stresses, MYB15 gene transcription was up-regulated, and the MYB15 protein interacted with ICE1 and bound to Myb recognition sequences in the promoters of CBF genes. The CBF genes activate many downstream genes that have been connected to freezing tolerance in plants [61]. PacMYBA, a sweet cherry R2R3-MYB transcription factor, enhanced salt stress tolerance and pathogen resistance in transgenic Arabidopsis plants. This increased stress tolerance may be due to increased anthocyanin accumulation [62]. 

Considerable research has indicated that heat shock proteins (Hsp) [63,64], as molecular chaperones, are involved in many biological activities by folding, transporting, translocating, assembling, or degrading client proteins [65,66]. In this study, the expression levels of *heat shock protein* (Solyc07g047790.3), *calcium-binding protein* (Solyc06g073830.1), and *pathogenesis-related protein 1* (Solyc00g174340.2) were also significantly higher under DL conditions rather than the CL condition. Calcium-binding protein and pathogenesis-related protein were induced by R2R3MYB transcription factors [67]. These results suggest that DL stimulates plants defense responses likely via R2R3MYB transcription factors in tomato plants. 

Hormones. Plant hormones induce plant growth and development in response to environmental signals [68,69]. Under DL conditions, four common DEGs were found to encode proteins related to plant hormones, including *auxin-regulated IAA2* (Solyc06g084070.3), *IAA-amido synthetase 3-9* (Solyc07g063850.3), *protein phosphatase 2c* (Solyc06g051940.3), and pathogenesis-related protein 1 (Solyc00g174340.2) (Figure 7). IAA affected the plant phenotype by regulating several genes [70,71], all of which control plant cell division and elongation. Light conditions controlled the elongation of stem cells by an auxin-responsive GH3 gene homologue [72]. In this experiment, the expression trends of auxin gene expression levels of tomato seedlings under different treatments were consistent with the elongation of both plant height and leaves. These results also imply that hormonal crosstalk plays a vital role in the effect of a DL environment on plant morphology [73]. 

Carbon metabolic process-related genes. Photosynthetic pigments form the beginning of reception, transferal and capture of light energy. The light-harvesting efficiency of the photosystem is assumed to be largely dependent on the size of its photosynthetic antenna [74], which is controlled by the biosynthesis of chlorophyll b [75,76]. Light-harvesting antenna systems play the dual role of gathering and dissipating light energy to transfer just enough light energy to the reaction centers. Chlorophyll b is synthesized from chlorophyll a by chlorophyllide a oxygenase (CAO) [77]. Phosphoenolpyruvate carboxylase (PEPC) is one of the CO_2_-fixing enzymes, which forms oxaloacetate from phosphoenolpyruvate (PEP) and bicarbonate (HCO3-), releasing inorganic phosphate (Pi) in the presence of Mg^2+^ or Mn^2+^ [78]. In the leaves of C3 plants, PEPC participates in a variety of biosynthetic pathways and in nitrogen assimilation, where it acts as anaplerotic to replenish the tricarboxylic acid (TCA) cycle [79]. PEPC is activated by glucose-6-phosphate and inhibited by L-malate or aspartic acid (Asp) [80,81]. Trehalose-6-phosphate (T6P), a crucial regulator of sugar metabolism, growth, and development is widely distributed in higher plants [82,83,84]. T6P not only acts as a signaling metabolite of starch synthesis [85], but also as an effector to inhibit the hexokinase and control glycolytic flux [86]. The biosynthesis of trehalose-6-phosphate involves glucose-6-phosphate and UDP-glucose by the enzyme trehalose-6-phosphate synthase (TPS) [87]. Moreover, trehalose-6-phosphate phosphatase (TPP) catalyzes the dephosphorylation of T6P to trehalose [87]. Trehalose plays an important role in protecting bioactive substances and cell structures under various stress environments, such as drought, freezing, high temperature, and salt [88,89,90,91]. Aldose 1-epimerase is a key regular of lactose metabolic processes. Lactose is hydrolyzed into D-glucose and β-D-galactose under β-galactosidases (= lactases). Then, β-D-galactose is catabolized via the Leloir pathway [92], but galactokinase, the first enzyme of the Leloir pathway, accepts only α-D-galactose and cannot act on the β-anomer in prokaryotes, yeasts, and mammals. In the present study, the down-regulations of *CAO* (Solyc11g012850.2), *PEPC* (Solyc04g006970.3), *TPS* (Solyc07g006500.3), and *aldose 1-epimerase* (Solyc02g087800.3) suggest that the DL light condition might not benefit chlorophyll b synthesis, CO_2_ assimilation, sugar formation, and sugar metabolism. 

The regulation among different pathways and their gene expression levels are important factors for plant development and interactions with the different light regimes. The changes in gene expressions varied in pathways of diterpenoid biosynthesis, sesquiterpenoid and triterpenoid biosynthesis, indicating the influences of light regimes on secondary metabolites. The gene expression levels of different DL regimes imply an influence of the circadian clock and the light intensity in coordinating the acclamatory responses of functionally-related genes.

Mitogen-activated protein kinasse signaling pathway. Mitogen-activated protein kinases (MAPKs) are serine/threonine protein kinases in eukaryotes. MAPKs are components of MAPK cascades, which are involved in the transduction of extracellular signals to intracellular targets and regulate the expression of special genes. MAPKs induce activation of defense responses in response to different extra cellular stimuli [93]. Recent findings clearly demonstrate that the auxin signal transduction is mediated by MAPKs [94]. Thus, auxin may promote plant defense responses by regulating the pathway of MAPK signaling, specially increasing the expression of *pathogenesis-related protein 1*. 

As a central mediator for the coordination of metabolism, the circadian clock in higher plants maintains homeostasis under a predictable, albeit changing, environment [57], which is involved in dynamic regulations of diverse physiological processes [13]. However, the highest light intensity of the treatments still did not reach the tomato light saturation point, which is limited by the number of lamps in the artificial growth box. Further investigations are needed to identify the key regulated metabolites and the relationships between transcriptome and metabolome [58]. Furthermore, the influence of light intensity distributions during a complete tomato life circle should be investigated, especially the key metabolic differences of tomato fruit quality at the red-ripe stage. 

## 5. Conclusions

DL regimes affected the early development stage of tomato plants at the morphological, photosynthetic, and transcriptional levels. It slowed the plant development and regulated many regulatory pathways, such as plant–pathogen genes, heat shock protein, lignin biosynthesis genes, and auxin-related genes. DL regimes also suppressed the expression of photosynthesis related genes, such as *chlorophyllide a oxygenase*, *phosphoenolpyruvate carboxylas,* and *trehalose-6-phosphate synthase 1*, resulting in a decrease of photosynthetic rate, especially in the M treatment, which had the highest light intensity during midday. This analysis also showed that light intensity regulated many circadian clock-related genes, which could be useful for the utilization of light source under artificial light sources. This study provides new morphological, photosynthetic, and transcriptional regulations that underlie light regimes for plant development.

## Figures and Tables

**Table 1:** Comparison of Pn, Gs, total chlorophyll content, Chl a/b, and leaf area in different light regimes. (*n* = 10 for Pn, Gs, and leaf area. *n* = 7 for total chlorophyll and Chl a/b.)

Treatments	Pn (μmol·m^-2^·s^-1^)	Gs (mmol·m^-2^·s^-1^)	Total Chlorophyll (mg g^-1^ FW)	Chl a/b	Leaf Area (cm^2^)
CL	4.94 ± 0.11 a	0.38 ± 0.03 a	2.75 ± 0.02 a	2.76 ± 0.01 c	123.70 ± 5.14 a
M	4.78 ± 0.13 ab	0.31 ± 0.01 ab	2.53 ± 0.05 b	2.83 ± 0.02 b	102.32 ± 4.15 b
A	4.71 ± 0.08 ab	0.38 ± 0.04 a	2.54 ± 0.04 b	2.82 ± 0.02 b	115.16 ± 2.33 ab
D	4.56 ± 0.08 b	0.29 ± 0.03 b	2.39 ± 0.08 b	2.87 ± 0.01 a	103.26 ± 4.74 b

Note: Net photosynthetic rate (Pn); stomatal conductance (Gs). Different lowercase letters indicate significant difference (*p* < 0.05).

## Supplementary Materials

The following are available online at https://www.mdpi.com/2073-4425/10/9/662/s1. Table S1. Summary of base quality before and after filtering and the number of mapped reads to the reference genome; Table S2. Differentially expressed genes (DEGs) between treatments and control; Table S3. All significantly expressed genes between treatment 1 and the control; Table S4. Significant DEGs between treatment 2 and control; Table S5. Significant DEGs between treatment 3 and control; Table S6. Significant DEGs between treatments 1 and 2; Table S7. Significant DEGs between treatments 1 and 3; Table S8. Significant DEGs between treatments 2 and 3
